# Effects of a Mindfulness Meditation Course on Learning and Cognitive Performance among University Students in Taiwan

**DOI:** 10.1155/2015/254358

**Published:** 2015-11-10

**Authors:** Ho-Hoi Ching, Malcolm Koo, Tsung-Huang Tsai, Chiu-Yuan Chen

**Affiliations:** ^1^Department of Natural Biotechnology, Nanhua University, Dalin, Chiayi 62249, Taiwan; ^2^Department of Medical Research, Dalin Tzu Chi Hospital, Buddhist Tzu Chi Medical Foundation, Dalin, Chiayi 62247, Taiwan; ^3^Dalla Lana School of Public Health, University of Toronto, Toronto, ON, Canada M5T 3M7; ^4^Department of Psychiatry, Dalin Tzu Chi Hospital, Buddhist Tzu Chi Medical Foundation, Dalin, Chiayi 62247, Taiwan; ^5^Research and Extension Center of Natural Healing Sciences, Nanhua University, Dalin, Chiayi 62249, Taiwan

## Abstract

Mindfulness training has recently gained much research interest because of its putative benefits for both mental and physical health. However, little is available in its effects on Asian students. Therefore, a quasi-experimental pre/posttest design was used to assess the effects of a one-semester mindfulness meditation course in 152 first-year Taiwanese university students and compared with 130 controls. The Chinese version of the College Learning Effectiveness Inventory (CLEI) and a computer software program focused on specific cognitive tasks were used for the evaluation. Results from the analysis of covariance revealed that while the score of the full CLEI scale was significantly higher in the intervention group compared with the control (*P* = 0.022), none of the comparisons between the nine CLEI subscales were significantly different between the two groups. For the computer cognitive tasks, the intervention group exhibited significantly better performance in the accuracy of the digital vigilance task (*P* = 0.048), choice reaction time (*P* = 0.004), spatial working memory (*P* = 0.042), and digital vigilance task reaction time (*P* = 0.004). This study showed that a one-semester mindfulness meditation course was able to improve learning effectiveness and both attention and memory aspects of cognitive performance among Taiwanese university students.

## 1. Introduction

Mindfulness can be considered as a meditation practice that cultivates present moment awareness [1]. The word mindfulness originates from the combination of two Pali words, Sati which means “awareness” and Samprajanya, “clear comprehension.” Mindfulness meditation aims to foster inner calmness and nonjudgment of the mind, which can help individuals to “acknowledge and accept as it is” in all aspects of daily life [2, 3]. A PubMed search of the term mindfulness revealed the following trend: 11 articles published between 1980 and 1989; 28 articles published between 1990 and 1999; 510 articles published between 2000 and 2010; and 2,263 articles published between 2011 and August 2015. Mindfulness meditation has been studied in a broad range of mental and physical health outcomes, such as major depression [4], cancer [5], HIV pathogenesis [6], multiple sclerosis [7], chronic low back pain [8], chronic insomnia [9], and chronic kidney disease [10].

In addition to its effects on pathogenesis, mindfulness meditation has also been evaluated regarding its putative beneficial role in improving attention [11, 12], cognition [13], cognitive flexibility [14, 15], and academic performance [16]. Nevertheless, to our knowledge, no studies have evaluated the effects of mindfulness meditation on Taiwanese university students. We took advantage of a new curriculum offered by a university in Taiwan in which a mindfulness meditation course is a mandatory credited course for all first-year students. The aim of this study was to explore the effects of a mindfulness meditation course on learning and cognitive performance among Taiwanese university students.

## 2. Methods

### 2.1. Study Design and Participants

This study was carried out in accordance with the protocol approved by the Institutional Review Board of the Buddhist Dalin Tzu Chi Hospital, Taiwan (number B10303014).

This intervention study used a quasi-experimental pre/posttest design. This study was conducted in a private university in south Taiwan where a one-semester course in mindfulness meditation is a part of the core curriculum for all first-year students. Since the course was offered in both the first and second semester and this study was conducted in the first semester of a school year, we recruited the students who were enrolled in the mindfulness meditation course (a total of 12 classes) as the intervention group and those who would take the course (another 12 classes) in the second semester as the control group.

After obtaining the approval from the course instructor, one of the study investigators (Ho-Hoi Ching) went to each of the 24 classes, that is, 12 classes of mindfulness meditation and 12 classes of physical exercise, to explain the research and invite participants.

A total of 359 students, 204 in the intervention group and 155 in the control group, consented to participate in the study. During the study period, 52 (25%) and 25 (16%) students in the intervention group and control group, respectively, dropped out from the study. Therefore, only 152 (75%) in the intervention group and 130 (84%) controls were included in the data analyses.

### 2.2. Intervention

The mindfulness meditation course consisted of 18 weekly 50-minute classes (a total of 15 hours) during September in 2014 and February 2015. The course content was modeled based on the concepts of mindfulness-based meditations with reference to traditional Buddhists context. Students in the mindfulness meditation course engaged in formal meditation practices such as mindful breathing, body scan, and eating and walking meditation. During class time, the focus was on formal practice but students were encouraged to apply mindfulness skills in their everyday life. Students were required to attend classes, to submit a practice diary, and to watch online course materials outside of the classes as part of their course assessment. The outline of the mindfulness meditation intervention is included in Table 1.

### 2.3. Measurement Tools

This study used the Chinese version of the College Learning Effectiveness Inventory (CLEI) and a set of computer cognitive tasks to measure the effects of mindfulness meditation intervention on students' learning and cognition (attention and working memory), respectively. The pretest and posttest were administrated to the students at week 3 and week 15, respectively.

### 2.4. College Learning Effectiveness Inventory

The College Learning Effectiveness Inventory (CLEI) is a valid and reliable assessment tool designed to measure college students' attitudes and behaviors that may impact learning and academic performance. The CLEI contains clearly definable and operational items to measure psychosocial factors including thoughts, feelings, or behaviors related to academic outcomes [17].

The CLEI was previously translated into Chinese and its psychometric properties were evaluated in 420 Taiwanese university students. A Cronbach alpha of 0.90 was obtained for the Chinese version of CLEI, indicating a good internal consistency. In addition, the 3-week test-retest study was conducted on 110 students and a test-retest reliability coefficient of 0.89 was obtained for the scale [18]. The scale consists of nine subscales with 38 questions using five-point Likert-scale responses from never to always. Negatively phrased items were reverse scored. The total scores range from 38 to 190 with a higher score representing better learning effectiveness.

The nine subscales of the Chinese version of CLEI are as follows. (1) Emotional satisfaction: it reflects the extent of students' interest in university life and emotional reactions, including those towards people and to the environment on campus. (2) Management (plan and time management): it reveals the ability to organize and time management when setting goals and make plans to carry out the necessary academic activities among college students. (3) Achievement: it reflects the degree of students in actively setting goals for themselves and trying to pursue success and also reflects students' own expectations for success. (4) Stress: it reflects students perceived pressure from schoolwork and time and whether they can properly handle stress from the environment and the academic needs. (5) Attention to study: it reveals the extent of university students for learning and academic focus. (6) Class communication: it includes communications between teachers and students in classroom activities, including verbal and nonverbal part. (7) Confidence: it reflects the faith and confidence demonstrated by students for their academic ability. (8) Involvement with college activity: it refers to belonging to certain organizations or participating in some activities within the campus environment, including social and academic activities. (9) Friendship: it refers to the interaction and friendship with friends, classmates, or other students when participating the campus community or activities, such as group assignments or studying together. In this study, the Chinese version of the CLEI was administered online using an open-source learning management system Moodle (https://moodle.org).

### 2.5. Computer Cognitive Tasks

The cognitive measurement computer software E-prime was used in this study to evaluate cognitive performance of attention and working memory [19]. Students were asked to complete it after they had finished the CLEI survey at both the pretest and posttest. The four cognitive tasks included in this study were digit vigilance tasks, choice reaction times, spatial working memory, and memory scanning tasks. The first two tasks were used to measure attention and the last two were used to measure memory. A basic description of these four tasks is as follows. (1) Digit vigilance task: a number (from 1 to 9 and 0) was displayed constantly on different places of the screen. If the appearing number matched with the number in the middle, the participant must press the “1” button as quickly as possible every time the digit in the center matched the one constantly displayed. If the appearing number did not match with the number in the middle, the participant had to press the “0” button. Accuracy of response (%) and reaction time (milliseconds) were recorded. A higher percentage in accuracy and a lower value in reaction time represent better performance. This task was designed to measure sustained attention. (2) Choice reaction time: either the letter X or the letter Y was presented on different parts of the screen. The participant must press the “1” for X or “0” button for Y as appropriate and as quickly as possible. There were 50 trials and the interval varied randomly between 1 and 2.5 seconds. Accuracy (%) and reaction time (milliseconds) were recorded. This task was designed to measure attention and vigilance. In the posttest, X and Y were changed to U and V to reduce learning effect. (3) Spatial working memory: a schematic picture of a house was presented for 5 seconds. The house had nine windows in a 3 × 3 pattern, 4 of which were illuminated. A series of 50 presentations of the same house in which just one window was illuminated follow, and the participant had to respond “1” if the window was one of the four lit in the original presentation or “0” if it was not. Reaction time and accuracy were recorded. This task was designed to measure memory. In the pretest, the color of the box was yellow and it was changed to red in the posttest. (4) Memory scanning task: five digits were presented singly at the rate of one per second for the participant to remember. A series of thirty digits was then presented. For each, the participant must press 1 or 0 according to whether the digit was thought to be one of the five presented initially. This was repeated three times using a different set of 5 digits on each occasion. Reaction time and accuracy were recorded. This task was also designed to measure memory.

### 2.6. Statistical Analysis

Descriptive statistics were used to examine the sample demographic data, including sex, religious affiliations, perceived health status, experience practicing mindfulness meditation, and college of study. The distributions of these variables in the intervention group and control were compared with chi-square test. Independent *t*-test was used to compare the posttest minus pretest (delta) scores between intervention group and control group. Analysis of covariance (ANCOVA) was used to compare the scores obtained from the full CLEI and each of its nine subscales between intervention group and control group, adjusted for the potential confounding effects of pretest scores, sex, college, and experience with mindfulness meditation. In addition, the accuracy and reaction time for the four computer cognitive tasks between the intervention group and control were each separately analyzed with ANCOVA. Least square means and their associated 95% confidence intervals were presented. All analyses were conducted using IBM SPSS Statistics software package, version 21.0 (IBM Corp., Armonk, NY, USA). A *P* < 0.05 was considered statistically significant.

## 3. Results

A total of 282 students, 152 in the intervention group and 130 in the control group, were included in the data analyses of the study. All participants were between 18 and 19 years of age and 61% were females. The distributions of their sex, religious affiliations, perceived health status, experience with mindfulness mediation, and the college of study were shown in Table 2. Except religious affiliations and perceived health status, the other factors were significantly different between the intervention group and control. These factors were included in the subsequent ANCOVA to adjust for their potential confounding effects.

The unadjusted means of CLEI and computer cognitive tasks at week 3 and week 15 for the intervention group and control group are shown in Table 3. No significant differences were observed in the delta scores of the scales between intervention group and control group.

Table 4 shows the least squares mean scores for the CLEI and computer cognitive tasks. While the score of the full CLEI scale was significantly higher in the intervention group compared with the control (*P* = 0.022), none of the comparisons between the nine CLEI subscales were significantly different between the two groups. For the computer cognitive tasks, the intervention group exhibited significantly better performance in the accuracy of the digital vigilance task (*P* = 0.048), choice reaction time (*P* = 0.004), and spatial working memory (*P* = 0.042) and also in the reaction time of the digital vigilance task (*P* = 0.004).

## 4. Discussion

In this quasi-experimental pre/posttest design study, the effects of a curriculum-based mindfulness meditation course on learning and cognitive performance among university students were investigated. Overall, we found that a one-semester mindfulness meditation course was able to significantly improve learning performance, as reflected by the least squares mean scores of the full CLEI scale (*P* = 0.022). However, none of nine subscales showed significant differences between the intervention group and control. Based on the literature [20], we anticipated that the stress subscale would have showed improvements among students with mindfulness meditation training. A literature review of 14 medical schools that taught mindfulness to medical students and residents suggested that mindfulness programs were able to decrease psychological distress [21]. A possible reason for the lack of significant effect on stress in the present study could be a regression to the mean effect where stress levels are high at the beginning of a school term but will naturally regress to a low level once students are familiar with university life, regardless of whether they have enrolled in the mindfulness meditation program. Another possible reason is that the mindfulness meditation program was offered as a mandatory course. Not all students were interested and engaged in using mindfulness meditation as a way to manage their stress levels. Our findings might be different if the program was offered as an elective course where enrolled students are more motivated. A 5-week mindfulness-based curriculum on teacher-ratings of student classroom behavior at a public elementary school showed improved classroom behavior such as paying attention, self-control, participation in activities, and caring/respect for others [22]. Nevertheless, no control group was used for a comparison in the study. The effects of mindfulness meditation on elementary school students and university students may be different and will require further research.

Regarding the effects on cognitive performance, the mindfulness meditation course was able to significantly improve the accuracy of digital vigilance task, choice reaction time, and spatial working memory as well as the reaction time in digital vigilance task. Improvements in attentional task performance were consistent with previous research [11, 12]. It is suggested that the process of mindfulness training can promote attentional stability by promoting a balance between a relaxed and vigilant state of mind and thereby enhancing cognition through a better ability to self-regulate emotions [23]. Moreover, mindfulness training may increase participants' cognitive performance by improving their mood and reducing mind wandering. Mind wandering and mood disturbances can negatively impact learning and memory [11]. A series of studies on short-term mindfulness-based intervention on undergraduate students suggested that the associated improvements in executive function are related to the neural circuitry specific to the anterior cingulate cortex and the autonomic nervous system [24].

A few limitations should be noted when interpreting the results of this study. First, the group allocation was not based on random assignment. Although the factors with uneven distribution between two groups were adjusted in ANCOVA, other unmeasured factors such as sleep quality and usage of caffeine beverages could potentially affect the results. Second, due to unforeseen computer issues, approximately half of the controls and a quarter of the participants in the intervention group were not able to complete the computer cognitive tasks as expected. Nevertheless, the computer issues should not be associated with the outcome. In fact, we used ANCOVA to compare the CLEI scores of the participants with missing data with those of participants without missing data on computer cognitive task. We found no significant differences between the two groups (*P* = 0.653), implying that the pattern of missing is random. Third, approximately 22% of the initial participants dropped out from the study. Fourth, we used a binary variable to adjust for the experience in practicing mindfulness meditation. Information on the length and frequency of practice was not ascertained and therefore cannot be further adjusted. Fifth, the *P* values obtained should be interpreted in view of the increased chance of type 1 error as a result of multiple comparisons of subscales of the CLEI and components of the computer cognitive task. Finally, the possibility of a placebo effect cannot be evaluated and future studies should consider the use of a sham meditation group [25].

In conclusion, findings from this study are consistent with the notion that attentional performance can be trained. A one-semester mindfulness meditation course was able to improve learning effectiveness and both attention and memory aspects of cognitive performance among university students. Effective learning and sustained attention and memory are important requirements for success and well-being in academic contexts. Incorporating a mindfulness meditation course in the curriculum may be a feasible approach to improve learning effectiveness and cognition performance in university students.

## Figures and Tables

**Table 1:** Outline of the mindfulness meditation intervention.

Week	Content
1	(i) Course orientation (ii) Basics of body scan (iii) Mindful eating

2	(i) Advanced body scan (ii) 3-minute breathing meditation (iii) Training attention and awareness through mindful breathing (iv) Basics of diary on mindfulness practice

3	(i) Walking meditation (live in the moment) (ii) Breathing techniques (iii) The classical basis of the mindfulness meditation course (iv) *Administration of the pretest (College Learning Effectiveness Inventory and computer cognitive tasks)*

4	Mindful meditations

5	(i) Mindfulness practice before sleeping (ii) Nonjudgmental versus judgmental

6	Mindfulness and the Noble Eightfold Path

7	(i) From mindfulness to “fullness of understanding” (ii) Mindfulness diary writing skills

8	(i) How do we know the world (ii) Mindfulness and health

9 (mid-term exam)	50-minute mindfulness meditation practice

10	(i) The beginner's mind (ii) Benefits of mindful speech

11	(i) Mindfulness interpersonal skills (ii) Basic use of dialectical behavior therapy

12	(i) Mindfulness and scientific research (ii) Mindfulness attitudes

13	Mindful living

14	(i) Purpose of behavior with “fullness of understanding” (ii) The use of the beginner's mind (iii) Balanced view of pros and cons

15	(i) Perspective views are decided by angles (ii) Investigating the filters of knowing (cognition) (iii) *Administration of the posttest (College Learning Effectiveness Inventory and computer cognitive tasks)*

16	Principle of not harming and principle of sincere treating

17	(i) Thoughts as thoughts, not necessarily the reality (ii) The use of mindfulness-based cognitive therapy (iii) The origin of life dependent (iv) Helpers also need mindfulness

18	50-minute mindfulness meditation practice

**Table 2:** Characteristics of the study participants (*N* = 282).

Variable	*n* (%)			Chi-square statistic	*P* value
	Total 282 (100)	Intervention group 152 (53.9)	Control group 130 (46.1)		
Sex				14.03	<0.001
Males	110 (39.0)	44 (28.9)	66 (50.8)
Females	172 (61.0)	108 (71.1)	64 (49.2)
Religious affiliation				2.69	0.441
None	145 (51.4)	79 (52.0)	66 (50.8)
Buddhism	43 (15.2)	25 (16.4)	18 (13.8)
Taoism	52 (18.4)	30 (19.7)	22 (16.9)
Others	42 (14.9)	18 (11.8)	24 (18.5)
Perceived health status				4.10	0.129
Healthy	159 (56.4)	94 (61.8)	65 (50.0)
Neutral	97 (34.4)	45 (29.6)	52 (40.0)
Unhealthy	26 (9.2)	13 (8.6)	13 (10.0)
Experience practicing mindfulness meditation				10.65	<0.001
Yes	170 (60.3)	105 (69.1)	65 (50.0)
No	112 (39.7)	47 (30.9)	65 (50.0)
College of study				60.76	<0.001
Management and social sciences	107 (37.9)	68 (44.7)	39 (30.0)
Humanities and arts	73 (25.9)	59 (38.8)	14 (10.8)
Science and technology	102 (36.2)	25 (16.4)	77 (59.2)

**Table 3:** Unadjusted means of College Learning Effectiveness Inventory (CLEI) and computer cognitive tasks at week 3 and week 15 for the intervention group and control group (*N* = 282).

Variable	Mean (standard deviation)						*P *value
	Intervention			Control			
	Pretest score (week 3)	Posttest score (week 15)	Δ score	Pretest score (week 3)	Posttest score (week 15)	Δ score	
College Learning Effectiveness Inventory (CLEI)		*n* = 152			*n* = 130		

*Full scale score*	127.3 ± 17.5	129.8 ± 17.5	2.5 ± 11.1	127.4 ± 19.2	127.7 ± 18.9	0.3 ± 9.2	0.063
(1) Emotional satisfaction score	17.4 ± 3.3	17.7 ± 3.3	0.3 ± 2.8	17.5 ± 3.5	17.7 ± 3.6	0.2 ± 2.1	0.641
(2) Management score	13.8 ± 2.7	14.0 ± 2.8	0.2 ± 2.2	13.2 ± 3.0	13.1 ± 2.8	−0.1 ± 1.8	0.267
(3) Achievement	17.0 ± 3.0	17.1 ± 3.1	0.1 ± 2.4	17.1 ± 3.3	17.1 ± 3.3	0.0 ± 2.0	0.601
(4) Stress	17.3 ± 2.7	17.2 ± 2.7	−0.1 ± 2.2	16.4 ± 3.4	16.5 ± 3.1	0.1 ± 2.0	0.541
(5) Attention to study	16.6 ± 3.3	16.5 ± 3.2	−0.1 ± 2.4	16.3 ± 3.4	16.4 ± 3.1	0.2 ± 2.0	0.340
(6) Class communication	12.0 ± 2.4	11.8 ± 2.5	−0.2 ± 1.7	12.5 ± 2.7	12.6 ± 2.5	0.1 ± 1.4	0.155
(7) Confidence	10.8 ± 2.1	10.8 ± 2.1	−0.1 ± 1.8	10.6 ± 2.6	10.8 ± 2.5	0.2 ± 1.7	0.246
(8) Involvement with college activity	12.8 ± 2.1	12.8 ± 2.1	0.0 ± 2.1	13.0 ± 2.6	13.0 ± 2.5	−0.1 ± 1.8	0.687
(9) Friendship	11.0 ± 2.0	10.6 ± 2.1	−0.3 ± 1.9	10.7 ± 2.3	10.8 ± 2.1	0.1 ± 1.6	0.050

Computer cognitive task							

(1) Digit vigilance task		*n* = 112			*n* = 66		
Accuracy (%)	81.8 ± 16.1	86.4 ± 11.8	4.6 ± 20.3	80.1 ± 14.5	81.6 ± 15.7	1.5 ± 21.9	0.348
Reaction time (msec)	32.8 ± 6.2	30.7 ± 4.6	−2.1 ± 8.2	33.7 ± 6.0	33.3 ± 7.9	−0.4 ± 10.5	0.247
(2) Choice reaction time		*n* = 112			*n* = 66		
Accuracy (%)	70.7 ± 17.7	75.0 ± 13.3	4.3 ± 22.2	65.4 ± 20.5	67.9 ± 20.6	2.5 ± 27.7	0.639
Reaction time (msec)	38.2 ± 9.1	38.4 ± 6.4	0.2 ± 10.4	37.6 ± 10.4	38.5 ± 9.2	0.9 ± 13.4	0.698
(3) Spatial working memory		*n* = 112			*n* = 66		
Accuracy (%)	83.6 ± 12.0	84.6 ± 6.5	1.0 ± 13.8	81.4 ± 12.9	81.3 ± 13.2	−0.2 ± 17.7	0.620
Reaction time (msec)	21.7 ± 5.5	20.9 ± 4.1	−0.8 ± 6.7	21.2 ± 6.7	22.0 ± 7.9	0.8 ± 10.7	0.220
(4) Memory scanning task		*n* = 109			*n* = 64		
Accuracy (%)	83.7 ± 14.7	84.6 ± 9.4	0.8 ± 17.4	82.3 ± 12.6	82.5 ± 12.9	0.1 ± 18.3	0.805
Reaction time (msec)	33.7 ± 6.5	32.0 ± 4.8	−1.8 ± 7.9	33.7 ± 5.2	32.8 ± 5.5	−0.8 ± 7.6	0.450

*P* values were calculated by independent *t*-test comparing Δ between intervention group and control group.

Δ: posttest values minus pretest values.

**Table 4:** Analysis of covariance (ANCOVA) for the effects of mindfulness meditation on College Learning Effectiveness Inventory (CLEI) and computer cognitive tasks (*N* = 282).

Variable	Least squares mean (95% confidence interval)^a^		*F* statistic (df)	*P* value
	Intervention	Control		
College Learning Effectiveness Inventory (CLEI)	*n* = 152	*n* = 130		

*Full scale score*	130.2 (128.5, 131.8)	127.2 (125.4, 129.0)	5.320 (1, 276)	0.022
(1) Emotional satisfaction score	17.6 (17.2, 18.0)	17.8 (17.3, 18.2)	0.227 (1, 276)	0.635
(2) Management score	13.7 (13.4, 14.0)	13.4 (13.0, 13.7)	1.753 (1, 276)	0.187
(3) Achievement	17.1 (16.8, 17.5)	17.1 (16.7, 17.5)	0.002 (1, 276)	0.964
(4) Stress	16.9 (16.6, 17.2)	16.9 (16.5, 17.2)	0.015 (1, 276)	0.904
(5) Attention to study	16.4 (16.0, 16.7)	16.5 (16.2, 16.9)	0.230 (1, 276)	0.632
(6) Class communication	12.0 (11.8, 12.3)	12.4 (12.1, 12.6)	2.717 (1, 276)	0.100
(7) Confidence	10.6 (10.4, 10.9)	10.9 (10.6, 11.2)	1.473 (1, 276)	0.226
(8) Involvement with college activity	12.8 (12.5, 13.1)	13.0 (12.7, 13.3)	0.742 (1, 276)	0.390
(9) Friendship	10.5 (10.2, 10.8)	10.9 (10.6, 11.2)	3.380 (1, 276)	0.067

Computer cognitive task				

(1) Digit vigilance task	*n* = 112	*n* = 66		
Accuracy (%)	86.2 (83.7, 88.7)	81.9 (78.5, 85.2)	3.982 (1, 172)	0.048
Reaction time (msec)	30.6 (29.4, 31.7)	33.4 (31.9, 35.0)	8.589 (1, 172)	0.004
(2) Choice reaction time	*n* = 112	*n* = 66		
Accuracy (%)	75.2 (72.1, 78.3)	67.6 (63.5, 71.6)	8.308 (1, 172)	0.004
Reaction time (msec)	38.1 (36.7, 39.5)	38.9 (37.0, 40.8)	0.427 (1, 172)	0.514
(3) Spatial working memory	*n* = 112	*n* = 66		
Accuracy (%)	84.5 (82.8, 86.3)	81.4 (79.0, 83.8)	4.210 (1, 172)	0.042
Reaction time (msec)	20.9 (19.8, 22.0)	22.0 (20.5, 23.4)	1.205 (1, 172)	0.274
(4) Memory scanning task	*n* = 109	*n* = 64		
Accuracy (%)	84.6 (82.5, 86.7)	82.3 (79.5, 85.1)	1.598 (1, 167)	0.208
Reaction time (msec)	32.0 (31.0, 33.0)	32.8 (31.5, 34.1)	0.974 (1, 167)	0.325

^a^Means were posttest scores adjusted for pretest scores, sex, college, and experience with mindfulness meditation.

msec: millisecond.
