# Corrigendum to “Effects of a Mindfulness Meditation Course on Learning and Cognitive Performance among University Students in Taiwan”

**DOI:** 10.1155/2016/5905180

**Published:** 2016-01-20

**Authors:** Hoi-Ching Ho, Malcolm Koo, Tsung-Huang Tsai, Chiu-Yuan Chen

**Affiliations:** ^1^Department of Natural Biotechnology, Nanhua University, Dalin, Chiayi 62249, Taiwan; ^2^Department of Medical Research, Dalin Tzu Chi Hospital, Buddhist Tzu Chi Medical Foundation, Dalin, Chiayi 62247, Taiwan; ^3^Dalla Lana School of Public Health, University of Toronto, Toronto, ON, Canada M5T 3M7; ^4^Department of Psychiatry, Dalin Tzu Chi Hospital, Buddhist Tzu Chi Medical Foundation, Dalin, Chiayi 62247, Taiwan; ^5^Research and Extension Center of Natural Healing Sciences, Nanhua University, Dalin, Chiayi 62249, Taiwan

The first author's name should be Hoi-Ching Ho in the paper titled “Effects of a Mindfulness Meditation Course on Learning and Cognitive Performance among University Students in Taiwan” [1].
